# Estimation of the Renal Function in Urinary Surgery

**Published:** 1910-09

**Authors:** 


**Estimation of the Renal Function in Urinary Surgery.** By J. W. THOMSON WALKER, M.B., C.M. (Edin.), F.R.C.S. Pp. xiv, 274. London: Cassell and Company, Limited. 1908.

The importance of discovering the degree of functional capacity possessed by the kidneys before operating upon any part of the urinary system is now well recognised. But it is doubtful if the general surgeons in this country even yet sufficiently use the best methods available for estimating renal adequacy. It is true that none of the available methods of investigation are perfect, but some of them are accurate enough and simple enough to merit routine application, and we welcome the appearance of the book before us, which will serve both as a stimulus and as a guide to the practice of such methods.

The work is in two parts. The first dealing with the estimation of the total renal function, and the second with the estimation of the functional capacity of each kidney separately.

In the earlier pages the symptoms and signs of renal inadequacy, many of which may easily be overlooked, are helpfully described. Then follows a description and critical survey of the methods, among others, of estimating renal function by cryoscopy of the blood and urine, by the elimination of methylene-blue and indigo-carmine, and by the excretion of sugar after injection of phloridzin.

In the second part the uses and technique of ureteric meatoscopy, of catheterisation of the ureters, and of intra-vesical separation of the urine from each kidney are fully described.

We are pleased to find that the author believes that the dangers alleged by some writers to attend catheterisation of the ureters are minimal if due care is exercised in carrying out the necessary manipulations, and that this, the most reliable of all methods for obtaining the urine from each kidney separately, without intermixture and without vesical contamination, is confidently recommended. We may state, without fear of contradiction by those who have seriously tried the method, that it is not difficult to accomplish in most cases, granted that the orifices of the ureters can be clearly brought into the field of vision.

The labour involved in carrying out many of the detailed investigations recorded in the pages of this work must have been great, and we note that the author recognises his indebtedness for much assistance to an "enthusiastic staff of house surgeons and nurses." We hope that those who endeavour to follow in the author's footsteps will meet with similar enthusiastic assistance ; but it is safe to say they will not do so unless they are enthusiastic themselves, and perhaps not even then. We consider that both personal and extra-personal enthusiasm will be required to follow out the excellent instructions given in the section on the practical application of the methods of investigating the functional capacity of each kidney. The instructions are to administer a diuretic and inject phloridzin, to catheterise the ureters, and to collect the urine from each kidney every quarter of an hour or every half hour for two or three hours, and to estimate the actual quantity and the percentage of the urea and sugar in each sample, and construct a chart showing the course of elimination of these substances.

We have nothing but praise for this book, as we consider it to be of conspicuous merit, and one which has filled a gap in English medical literature.

